# 8-Benz­yloxy-2-methyl-3-(2-methyl­phenyl)quinazolin-4(3*H*)-one

**DOI:** 10.1107/S1600536812007362

**Published:** 2012-02-29

**Authors:** Adel S. El-Azab, Alaa A.-M. Abdel-Aziz, Seik Weng Ng, Edward R. T. Tiekink

**Affiliations:** a Department of Pharmaceutical Chemistry, College of Pharmacy, King Saud University, Riyadh 11451, Saudi Arabia; b Department of Organic Chemistry, Faculty of Pharmacy, Al-Azhar University, Cairo 11884, Egypt; c Department of Medicinal Chemistry, Faculty of Pharmacy, University of Mansoura, Mansoura 35516, Egypt; d Department of Chemistry, University of Malaya, 50603 Kuala Lumpur, Malaysia; e Chemistry Department, Faculty of Science, King Abdulaziz University, PO Box 80203 Jeddah, Saudi Arabia

## Abstract

In the title methaqua­lone analogue, C_23_H_20_N_2_O_2_, the planes of the terminal aromatic rings [dihedral angle between them = 64.52 (7)°] approximately face the fused-ring methyl group and both are twisted with respect to the pyrimidine plane (r.m.s. deviation = 0.028 Å), forming dihedral angles of 86.9 (3) (with the 2-tolyl ring) and 65.57 (7)°. The 2-tolyl residue is disordered over two almost coplanar but opposite orientations with the major component having a site-occupancy factor of 0.893 (3). The three-dimensional crystal packing is consolidated by C—H⋯O, C—H⋯π and π–π [2-tol­yl–2-tolyl centroid–centroid distance = 3.8099 (6) Å] inter­actions.

## Related literature

For recent studies on the synthesis, drug discovery and crystal structures of quinazoline-4(3*H*)-one derivatives, see: El-Azab *et al.* (2010 ▶, 2012 ▶). For the anti-convulsant activity of the title methaqua­lone analogue, see: El-Azab *et al.* (2011 ▶). For a related structure, see: Stephenson *et al.* (2011 ▶).
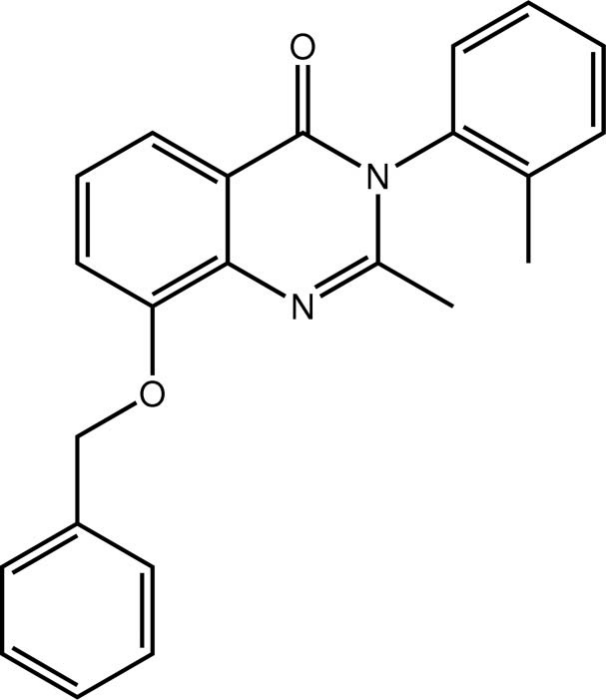


## Experimental

### 

#### Crystal data

C_23_H_20_N_2_O_2_
*M* *_r_* = 356.41
Monoclinic, 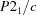

*a* = 18.2611 (3) Å
*b* = 7.6266 (1) Å
*c* = 13.2148 (2) Å
β = 91.094 (2)°
*V* = 1840.09 (5) Å^3^
*Z* = 4
Cu *K*α radiation
μ = 0.66 mm^−1^
*T* = 100 K
0.35 × 0.30 × 0.25 mm

#### Data collection

Agilent SuperNova Dual diffractometer with an Atlas detector
Absorption correction: multi-scan (*CrysAlis PRO*; Agilent, 2011 ▶) *T*_min_ = 0.967, *T*_max_ = 0.998
7568 measured reflections
3775 independent reflections
3559 reflections with *I* > 2σ(*I*)
*R*_int_ = 0.015

#### Refinement

*R*[*F* ^2^ > 2σ(*F* ^2^)] = 0.048
*wR*(*F* ^2^) = 0.121
*S* = 1.00
3775 reflections
245 parameters
43 restraints
H-atom parameters constrained
Δρ_max_ = 0.44 e Å^−3^
Δρ_min_ = −0.39 e Å^−3^

### 

Data collection: *CrysAlis PRO* (Agilent, 2011 ▶); cell refinement: *CrysAlis PRO*; data reduction: *CrysAlis PRO*; program(s) used to solve structure: *SHELXS97* (Sheldrick, 2008 ▶); program(s) used to refine structure: *SHELXL97* (Sheldrick, 2008 ▶); molecular graphics: *ORTEP-3* (Farrugia, 1997 ▶) and *DIAMOND* (Brandenburg, 2006 ▶); software used to prepare material for publication: *publCIF* (Westrip, 2010 ▶).

## Supplementary Material

Crystal structure: contains datablock(s) global, I. DOI: 10.1107/S1600536812007362/hb6643sup1.cif

Structure factors: contains datablock(s) I. DOI: 10.1107/S1600536812007362/hb6643Isup2.hkl

Supplementary material file. DOI: 10.1107/S1600536812007362/hb6643Isup3.cml

Additional supplementary materials:  crystallographic information; 3D view; checkCIF report

## Figures and Tables

**Table 1:** Hydrogen-bond geometry (Å, °) *Cg*1 is the centroid of the C18–C23 ring.

*D*—H⋯*A*	*D*—H	H⋯*A*	*D*⋯*A*	*D*—H⋯*A*
C13—H13⋯O1^i^	0.95	2.54	3.3250 (15)	140
C17—H17*B*⋯*Cg*1^ii^	0.99	2.62	3.5086 (16)	150
C22—H22⋯*Cg*1^iii^	0.95	2.77	3.5692 (16)	143

Symmetry codes: (i) 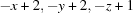; (ii) 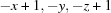; (iii) 
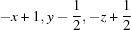
.

**Fig. 1.**
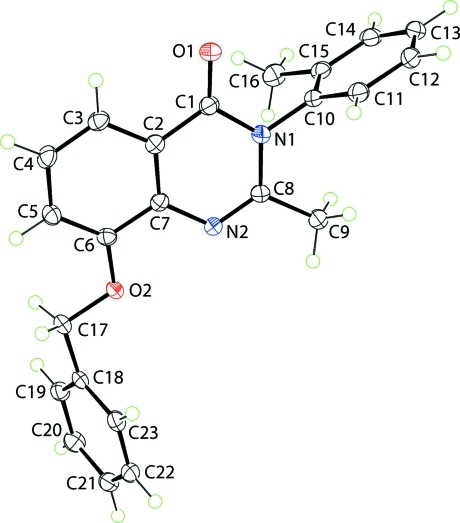
The molecular structure of (I) showing displacement ellipsoids at the 50% probability level. The minor disorder component of the toluyl ring is not shown.

**Fig. 2.**
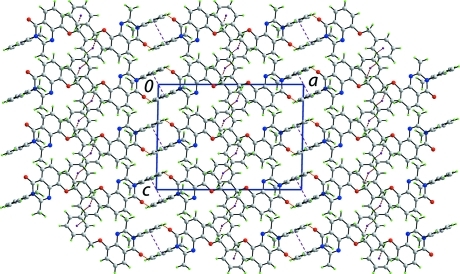
A view in projection down the b axis of the unit-cell contents of (I). The C—H···O, C—H···π and π–π interactions are shown as orange, bronw and purple dashed lines, respectively.

## supplementary crystallographic information

### Comment

Quinazoline-4(3*H*)-one derivatives are known for their various biological activities (El-Azab *et al.*, 2012, 2010). The title methaqualone analogue, 8-benzyloxy-2-methyl-3-(2-methylphenyl)-4(3*H*)-quinazolinone (I), has been investigated previously for its anti-convulsant activity (El-Azab *et al.*, 2011). Herein, its crystal and molecular structure is described. A related structure with a similar conformation has been reported recently (Stephenson *et al.*, 2011).

In (I), Fig. 1, the 2-tolyl group is orthogonal to the pyrimidine ring [r.m.s. deviation = 0.028 Å] forming a dihedral angle of 87.86 (6)°; the equivalent angle for the minor component of the disordered 2-tolyl ring = 86.9 (3)°. The phenyl ring of the benzyloxy residue is also twisted out of this plane, forming a dihedral angle of 65.57 (7)°. To a first approximation the planes through the terminal rings face towards the methyl group with the dihedral angle between them being 64.52 (7)°.

In the crystal packing, C—H···O and C—H···π interactions, Table 1, combine with π–π [ring centroid(2-tolyl)-to-centroid(2-tolyl)^i^ distance = 3.8099 (6) Å for symmetry operation *i*: 2 - *x*, 2 - *y*, 1 - *z*] interactions to consolidate molecules into the three-dimensional architecture, Fig. 2.

### Experimental

A mixture of 8-hydroxymethaqualone (532 mg, 2 mmol) and benzyl chloride (266 mg, 2.1 mmol) in acetone (15 ml) containing anhydrous potassium carbonate (415 mg, 3 mmol) was heated under reflux for 10 h. The reaction mixture was filtered while hot, the solvent was removed under reduced pressure, and the solid obtained was dried and recrystallized from AcOH as colourless prisms. Yield 86%; *M*.pt: 449–451 K. ^1^H NMR (500 MHz, CDCl_3_): δ 7.89 (d, 1H, J = 8.0 Hz), 7.53 (d, 2H, J = 7.0 Hz), 7.41–7.31 (m, 7H), 7.22 (d, 1H, J = 8.0 Hz), 7.17 (d, 1H, J = 7.5 Hz), 5.40 (s, 2H), 2.27 (s, 3H), 2.15 (s, 3H). ^13^C NMR (CDCl_3_): δ 17.4, 24.3, 71.3, 117.1, 119.0, 122.1, 126.6, 127.0, 127.6, 127.9, 128.0, 128.6, 129.5, 131.5, 135.3, 136.8, 137.0, 138.8, 153.3, 153.6, 161.5 p.p.m.. MS (70 eV): m/*z* = 356.

### Refinement

Carbon-bound H-atoms were placed in calculated positions [C—H = 0.95 to 0.99 Å, *U*_iso_(H) = 1.2 to 1.5*U*_eq_(C)] and were included in the refinement in the riding model approximation. The 2-tolyl ring is disordered over two co-planar positions with the methyl group being orientated in opposite directions. The benzene rings were refined as rigid hexagons with C—C = 1.39 Å. The major component refined to a site occupancy factor of 0.893 (3). The C—C distances were restrained to ±0.01 Å, of each other. The anisotropic displacement parameters were restrained to be nearly isotropic, and those of the primed carbon atoms were equated to those of unprimed atoms.

### Crystal data

**Table:**

C_23_H_20_N_2_O_2_	*F*(000) = 752
*M**_r_* = 356.41	*D*_x_ = 1.287 Mg m^−^^3^
Monoclinic, *P*2_1_/*c*	Cu *K*α radiation, λ = 1.54184 Å
Hall symbol: -P 2ybc	Cell parameters from 4269 reflections
*a* = 18.2611 (3) Å	θ = 3.3–76.3°
*b* = 7.6266 (1) Å	µ = 0.66 mm^−^^1^
*c* = 13.2148 (2) Å	*T* = 100 K
β = 91.094 (2)°	Prism, colourless
*V* = 1840.09 (5) Å^3^	0.35 × 0.30 × 0.25 mm
*Z* = 4	

### Data collection

**Table:**

Agilent SuperNova Dual diffractometer with an Atlas detector	3775 independent reflections
Radiation source: SuperNova (Cu) X-ray Source	3559 reflections with *I* > 2σ(*I*)
Mirror monochromator	*R*_int_ = 0.015
Detector resolution: 10.4041 pixels mm^-1^	θ_max_ = 76.5°, θ_min_ = 4.8°
ω scan	*h* = −15→22
Absorption correction: multi-scan (*CrysAlis PRO*; Agilent, 2011)	*k* = −9→9
*T*_min_ = 0.967, *T*_max_ = 0.998	*l* = −16→15
7568 measured reflections	

### Refinement

**Table:**

Refinement on *F*^2^	Primary atom site location: structure-invariant direct methods
Least-squares matrix: full	Secondary atom site location: difference Fourier map
*R*[*F*^2^ > 2σ(*F*^2^)] = 0.048	Hydrogen site location: inferred from neighbouring sites
*wR*(*F*^2^) = 0.121	H-atom parameters constrained
*S* = 1.00	*w* = 1/[σ^2^(*F*_o_^2^) + (0.0589*P*)^2^ + 1.3045*P*] where *P* = (*F*_o_^2^ + 2*F*_c_^2^)/3
3775 reflections	(Δ/σ)_max_ < 0.001
245 parameters	Δρ_max_ = 0.44 e Å^−^^3^
43 restraints	Δρ_min_ = −0.39 e Å^−^^3^

### Special details

**Table:**

Geometry. All e.s.d.'s (except the e.s.d. in the dihedral angle between two l.s. planes) are estimated using the full covariance matrix. The cell e.s.d.'s are taken into account individually in the estimation of e.s.d.'s in distances, angles and torsion angles; correlations between e.s.d.'s in cell parameters are only used when they are defined by crystal symmetry. An approximate (isotropic) treatment of cell e.s.d.'s is used for estimating e.s.d.'s involving l.s. planes.
Refinement. Refinement of *F*^2^ against ALL reflections. The weighted *R*-factor *wR* and goodness of fit *S* are based on *F*^2^, conventional *R*-factors *R* are based on *F*, with *F* set to zero for negative *F*^2^. The threshold expression of *F*^2^ > σ(*F*^2^) is used only for calculating *R*-factors(gt) *etc*. and is not relevant to the choice of reflections for refinement. *R*-factors based on *F*^2^ are statistically about twice as large as those based on *F*, and *R*- factors based on ALL data will be even larger.

### Fractional atomic coordinates and isotropic or equivalent isotropic displacement parameters (Å^2^)

**Table:**

	*x*	*y*	*z*	*U*_iso_*/*U*_eq_	Occ. (<1)
O1	0.90142 (6)	0.63543 (15)	0.61822 (8)	0.0249 (3)	
O2	0.61946 (5)	0.31632 (14)	0.48348 (7)	0.0199 (2)	
N1	0.84939 (7)	0.63272 (18)	0.45982 (9)	0.0226 (3)	
N2	0.73780 (6)	0.48656 (16)	0.42164 (9)	0.0182 (3)	
C1	0.85181 (8)	0.58540 (19)	0.56229 (10)	0.0194 (3)	
C2	0.79063 (7)	0.47545 (18)	0.59336 (10)	0.0175 (3)	
C3	0.78755 (8)	0.41602 (19)	0.69377 (11)	0.0209 (3)	
H3	0.8263	0.4407	0.7406	0.025*	
C4	0.72731 (8)	0.3212 (2)	0.72336 (11)	0.0230 (3)	
H4	0.7248	0.2795	0.7910	0.028*	
C5	0.66974 (8)	0.28556 (19)	0.65475 (11)	0.0214 (3)	
H5	0.6282	0.2218	0.6766	0.026*	
C6	0.67287 (8)	0.34249 (18)	0.55538 (10)	0.0180 (3)	
C7	0.73479 (7)	0.43755 (18)	0.52274 (10)	0.0166 (3)	
C8	0.79355 (8)	0.5776 (2)	0.39407 (11)	0.0208 (3)	
C9	0.80026 (9)	0.6254 (3)	0.28457 (11)	0.0311 (4)	
H9A	0.7617	0.5662	0.2449	0.047*	
H9B	0.8483	0.5885	0.2605	0.047*	
H9C	0.7952	0.7526	0.2767	0.047*	
C10	0.90451 (5)	0.75133 (12)	0.42618 (8)	0.0187 (4)	0.893 (3)
C11	0.97023 (6)	0.69096 (11)	0.38786 (9)	0.0231 (4)	0.893 (3)
H11	0.9798	0.5686	0.3852	0.028*	0.893 (3)
C12	1.02189 (5)	0.80957 (15)	0.35339 (9)	0.0275 (4)	0.893 (3)
H12	1.0668	0.7683	0.3272	0.033*	0.893 (3)
C13	1.00783 (5)	0.98854 (14)	0.35724 (9)	0.0288 (4)	0.893 (3)
H13	1.0431	1.0696	0.3337	0.035*	0.893 (3)
C14	0.94210 (6)	1.04890 (10)	0.39556 (9)	0.0263 (4)	0.893 (3)
H14	0.9325	1.1712	0.3982	0.032*	0.893 (3)
C15	0.89044 (5)	0.93030 (14)	0.43003 (8)	0.0210 (4)	0.893 (3)
C16	0.81947 (10)	0.9980 (2)	0.47494 (14)	0.0272 (4)	0.893 (3)
H16A	0.8183	1.1263	0.4706	0.041*	0.893 (3)
H16B	0.8170	0.9621	0.5460	0.041*	0.893 (3)
H16C	0.7776	0.9491	0.4371	0.041*	0.893 (3)
C10'	0.8800 (5)	0.8186 (10)	0.4385 (7)	0.0187 (4)	0.107 (3)
C11'	0.8473 (4)	0.9812 (12)	0.4522 (7)	0.0231 (4)	0.107 (3)
H11'	0.7997	0.9884	0.4796	0.028*	0.107 (3)
C12'	0.8843 (5)	1.1334 (9)	0.4260 (8)	0.0275 (4)	0.107 (3)
H12'	0.8620	1.2445	0.4354	0.033*	0.107 (3)
C13'	0.9540 (5)	1.1229 (10)	0.3860 (8)	0.0288 (4)	0.107 (3)
H13'	0.9793	1.2269	0.3681	0.035*	0.107 (3)
C14'	0.9867 (4)	0.9603 (13)	0.3722 (8)	0.0263 (4)	0.107 (3)
H14'	1.0344	0.9531	0.3449	0.032*	0.107 (3)
C15'	0.9497 (5)	0.8081 (10)	0.3984 (7)	0.0210 (4)	0.107 (3)
C16'	0.9817 (12)	0.638 (3)	0.3910 (16)	0.0272 (4)	0.107 (3)
H16D	1.0309	0.6480	0.3631	0.041*	0.107 (3)
H16E	0.9512	0.5644	0.3465	0.041*	0.107 (3)
H16F	0.9850	0.5846	0.4584	0.041*	0.107 (3)
C17	0.55629 (8)	0.2214 (2)	0.51593 (11)	0.0215 (3)	
H17A	0.5314	0.2872	0.5699	0.026*	
H17B	0.5712	0.1057	0.5433	0.026*	
C18	0.50526 (8)	0.19755 (18)	0.42653 (11)	0.0190 (3)	
C19	0.43504 (8)	0.26836 (19)	0.42639 (11)	0.0223 (3)	
H19	0.4197	0.3369	0.4822	0.027*	
C20	0.38705 (8)	0.2392 (2)	0.34480 (12)	0.0250 (3)	
H20	0.3392	0.2882	0.3451	0.030*	
C21	0.40895 (8)	0.1388 (2)	0.26318 (11)	0.0238 (3)	
H21	0.3762	0.1183	0.2078	0.029*	
C22	0.47930 (8)	0.0684 (2)	0.26299 (11)	0.0226 (3)	
H22	0.4945	−0.0002	0.2072	0.027*	
C23	0.52728 (8)	0.09802 (19)	0.34386 (11)	0.0207 (3)	
H23	0.5753	0.0503	0.3430	0.025*	

### Atomic displacement parameters (Å^2^)

**Table:**

	*U*^11^	*U*^22^	*U*^33^	*U*^12^	*U*^13^	*U*^23^
O1	0.0211 (5)	0.0330 (6)	0.0204 (5)	−0.0064 (4)	−0.0038 (4)	−0.0010 (4)
O2	0.0192 (5)	0.0221 (5)	0.0185 (5)	−0.0068 (4)	−0.0001 (4)	0.0020 (4)
N1	0.0210 (6)	0.0291 (7)	0.0176 (6)	−0.0074 (5)	−0.0013 (5)	0.0010 (5)
N2	0.0185 (6)	0.0198 (6)	0.0163 (5)	−0.0020 (5)	0.0004 (4)	−0.0005 (5)
C1	0.0193 (6)	0.0213 (7)	0.0175 (6)	0.0001 (5)	−0.0004 (5)	−0.0013 (5)
C2	0.0184 (6)	0.0159 (6)	0.0183 (6)	0.0019 (5)	0.0006 (5)	−0.0008 (5)
C3	0.0242 (7)	0.0204 (7)	0.0179 (7)	0.0012 (6)	−0.0032 (5)	0.0010 (5)
C4	0.0299 (8)	0.0218 (7)	0.0173 (6)	0.0003 (6)	−0.0002 (6)	0.0047 (6)
C5	0.0242 (7)	0.0182 (7)	0.0218 (7)	−0.0032 (6)	0.0024 (6)	0.0021 (5)
C6	0.0197 (7)	0.0154 (6)	0.0189 (6)	−0.0005 (5)	−0.0008 (5)	−0.0015 (5)
C7	0.0192 (6)	0.0143 (6)	0.0163 (6)	0.0017 (5)	0.0007 (5)	−0.0007 (5)
C8	0.0200 (7)	0.0245 (7)	0.0176 (7)	−0.0044 (6)	−0.0017 (5)	−0.0002 (5)
C9	0.0302 (8)	0.0455 (10)	0.0176 (7)	−0.0165 (7)	−0.0025 (6)	0.0032 (7)
C10	0.0171 (8)	0.0232 (9)	0.0157 (7)	−0.0025 (6)	−0.0008 (6)	−0.0019 (6)
C11	0.0201 (9)	0.0283 (11)	0.0209 (8)	−0.0011 (8)	0.0000 (6)	−0.0036 (8)
C12	0.0183 (8)	0.0437 (11)	0.0204 (8)	−0.0057 (7)	0.0017 (6)	−0.0031 (7)
C13	0.0285 (9)	0.0391 (11)	0.0187 (8)	−0.0159 (8)	−0.0024 (6)	0.0024 (7)
C14	0.0329 (9)	0.0265 (9)	0.0193 (8)	−0.0094 (7)	−0.0059 (6)	0.0014 (7)
C15	0.0209 (8)	0.0254 (8)	0.0165 (7)	−0.0004 (7)	−0.0033 (6)	−0.0012 (6)
C16	0.0284 (9)	0.0250 (9)	0.0284 (9)	0.0046 (7)	0.0010 (7)	−0.0032 (7)
C10'	0.0171 (8)	0.0232 (9)	0.0157 (7)	−0.0025 (6)	−0.0008 (6)	−0.0019 (6)
C11'	0.0201 (9)	0.0283 (11)	0.0209 (8)	−0.0011 (8)	0.0000 (6)	−0.0036 (8)
C12'	0.0183 (8)	0.0437 (11)	0.0204 (8)	−0.0057 (7)	0.0017 (6)	−0.0031 (7)
C13'	0.0285 (9)	0.0391 (11)	0.0187 (8)	−0.0159 (8)	−0.0024 (6)	0.0024 (7)
C14'	0.0329 (9)	0.0265 (9)	0.0193 (8)	−0.0094 (7)	−0.0059 (6)	0.0014 (7)
C15'	0.0209 (8)	0.0254 (8)	0.0165 (7)	−0.0004 (7)	−0.0033 (6)	−0.0012 (6)
C16'	0.0284 (9)	0.0250 (9)	0.0284 (9)	0.0046 (7)	0.0010 (7)	−0.0032 (7)
C17	0.0211 (7)	0.0219 (7)	0.0217 (7)	−0.0072 (6)	0.0028 (5)	0.0011 (6)
C18	0.0204 (7)	0.0160 (6)	0.0206 (7)	−0.0059 (5)	0.0029 (5)	0.0016 (5)
C19	0.0231 (7)	0.0191 (7)	0.0248 (7)	−0.0020 (6)	0.0046 (6)	−0.0029 (6)
C20	0.0205 (7)	0.0238 (7)	0.0307 (8)	0.0007 (6)	0.0007 (6)	0.0001 (6)
C21	0.0251 (7)	0.0231 (7)	0.0231 (7)	−0.0052 (6)	−0.0018 (6)	0.0014 (6)
C22	0.0268 (7)	0.0198 (7)	0.0212 (7)	−0.0037 (6)	0.0048 (6)	−0.0016 (6)
C23	0.0188 (6)	0.0199 (7)	0.0237 (7)	−0.0015 (5)	0.0045 (5)	0.0010 (6)

### Geometric parameters (Å, º)

**Table:**

O1—C1	1.2195 (18)	C15—C16	1.5259 (19)
O2—C6	1.3632 (17)	C16—H16A	0.9800
O2—C17	1.4347 (16)	C16—H16B	0.9800
N1—C8	1.3919 (18)	C16—H16C	0.9800
N1—C1	1.4012 (18)	C10'—C11'	1.3900
N1—C10	1.4304 (14)	C10'—C15'	1.3900
N1—C10'	1.552 (6)	C11'—C12'	1.3900
N2—C8	1.2906 (19)	C11'—H11'	0.9500
N2—C7	1.3893 (17)	C12'—C13'	1.3900
C1—C2	1.4620 (19)	C12'—H12'	0.9500
C2—C7	1.3993 (19)	C13'—C14'	1.3900
C2—C3	1.4043 (19)	C13'—H13'	0.9500
C3—C4	1.379 (2)	C14'—C15'	1.3900
C3—H3	0.9500	C14'—H14'	0.9500
C4—C5	1.402 (2)	C15'—C16'	1.427 (19)
C4—H4	0.9500	C16'—H16D	0.9800
C5—C6	1.385 (2)	C16'—H16E	0.9800
C5—H5	0.9500	C16'—H16F	0.9800
C6—C7	1.4173 (19)	C17—C18	1.502 (2)
C8—C9	1.499 (2)	C17—H17A	0.9900
C9—H9A	0.9800	C17—H17B	0.9900
C9—H9B	0.9800	C18—C19	1.391 (2)
C9—H9C	0.9800	C18—C23	1.396 (2)
C10—C11	1.3900	C19—C20	1.394 (2)
C10—C15	1.3900	C19—H19	0.9500
C11—C12	1.3900	C20—C21	1.388 (2)
C11—H11	0.9500	C20—H20	0.9500
C12—C13	1.3900	C21—C22	1.392 (2)
C12—H12	0.9500	C21—H21	0.9500
C13—C14	1.3900	C22—C23	1.387 (2)
C13—H13	0.9500	C22—H22	0.9500
C14—C15	1.3900	C23—H23	0.9500
C14—H14	0.9500		
			
C6—O2—C17	115.74 (11)	C15—C14—H14	120.0
C8—N1—C1	122.33 (12)	C14—C15—C10	120.0
C8—N1—C10	120.55 (11)	C14—C15—C16	119.61 (10)
C1—N1—C10	116.99 (11)	C10—C15—C16	120.37 (10)
C8—N1—C10'	115.1 (4)	C11'—C10'—C15'	120.0
C1—N1—C10'	114.0 (4)	C11'—C10'—N1	129.3 (6)
C10—N1—C10'	26.8 (3)	C15'—C10'—N1	110.6 (6)
C8—N2—C7	117.53 (12)	C12'—C11'—C10'	120.0
O1—C1—N1	120.96 (13)	C12'—C11'—H11'	120.0
O1—C1—C2	124.90 (13)	C10'—C11'—H11'	120.0
N1—C1—C2	114.14 (12)	C11'—C12'—C13'	120.0
C7—C2—C3	121.45 (13)	C11'—C12'—H12'	120.0
C7—C2—C1	118.83 (12)	C13'—C12'—H12'	120.0
C3—C2—C1	119.67 (13)	C14'—C13'—C12'	120.0
C4—C3—C2	118.92 (13)	C14'—C13'—H13'	120.0
C4—C3—H3	120.5	C12'—C13'—H13'	120.0
C2—C3—H3	120.5	C13'—C14'—C15'	120.0
C3—C4—C5	120.71 (13)	C13'—C14'—H14'	120.0
C3—C4—H4	119.6	C15'—C14'—H14'	120.0
C5—C4—H4	119.6	C14'—C15'—C10'	120.0
C6—C5—C4	120.56 (13)	C14'—C15'—C16'	122.7 (11)
C6—C5—H5	119.7	C10'—C15'—C16'	117.2 (11)
C4—C5—H5	119.7	C15'—C16'—H16D	109.5
O2—C6—C5	124.95 (13)	C15'—C16'—H16E	109.5
O2—C6—C7	115.28 (12)	H16D—C16'—H16E	109.5
C5—C6—C7	119.76 (13)	C15'—C16'—H16F	109.5
N2—C7—C2	122.95 (12)	H16D—C16'—H16F	109.5
N2—C7—C6	118.47 (12)	H16E—C16'—H16F	109.5
C2—C7—C6	118.56 (12)	O2—C17—C18	108.44 (11)
N2—C8—N1	123.95 (13)	O2—C17—H17A	110.0
N2—C8—C9	118.85 (13)	C18—C17—H17A	110.0
N1—C8—C9	117.18 (13)	O2—C17—H17B	110.0
C8—C9—H9A	109.5	C18—C17—H17B	110.0
C8—C9—H9B	109.5	H17A—C17—H17B	108.4
H9A—C9—H9B	109.5	C19—C18—C23	119.29 (14)
C8—C9—H9C	109.5	C19—C18—C17	120.81 (13)
H9A—C9—H9C	109.5	C23—C18—C17	119.86 (13)
H9B—C9—H9C	109.5	C18—C19—C20	120.31 (14)
C11—C10—C15	120.0	C18—C19—H19	119.8
C11—C10—N1	121.43 (9)	C20—C19—H19	119.8
C15—C10—N1	118.55 (9)	C21—C20—C19	120.19 (14)
C10—C11—C12	120.0	C21—C20—H20	119.9
C10—C11—H11	120.0	C19—C20—H20	119.9
C12—C11—H11	120.0	C20—C21—C22	119.59 (14)
C11—C12—C13	120.0	C20—C21—H21	120.2
C11—C12—H12	120.0	C22—C21—H21	120.2
C13—C12—H12	120.0	C23—C22—C21	120.33 (14)
C14—C13—C12	120.0	C23—C22—H22	119.8
C14—C13—H13	120.0	C21—C22—H22	119.8
C12—C13—H13	120.0	C22—C23—C18	120.28 (13)
C13—C14—C15	120.0	C22—C23—H23	119.9
C13—C14—H14	120.0	C18—C23—H23	119.9
			
C8—N1—C1—O1	179.94 (14)	C10'—N1—C10—C15	0.0 (8)
C10—N1—C1—O1	−4.3 (2)	C15—C10—C11—C12	0.0
C10'—N1—C1—O1	−33.8 (4)	N1—C10—C11—C12	178.35 (11)
C8—N1—C1—C2	−0.5 (2)	C10—C11—C12—C13	0.0
C10—N1—C1—C2	175.30 (11)	C11—C12—C13—C14	0.0
C10'—N1—C1—C2	145.7 (4)	C12—C13—C14—C15	0.0
O1—C1—C2—C7	175.70 (14)	C13—C14—C15—C10	0.0
N1—C1—C2—C7	−3.84 (19)	C13—C14—C15—C16	178.14 (11)
O1—C1—C2—C3	−1.8 (2)	C11—C10—C15—C14	0.0
N1—C1—C2—C3	178.64 (13)	N1—C10—C15—C14	−178.40 (10)
C7—C2—C3—C4	−1.3 (2)	C11—C10—C15—C16	−178.12 (11)
C1—C2—C3—C4	176.19 (13)	N1—C10—C15—C16	3.48 (13)
C2—C3—C4—C5	−0.5 (2)	C8—N1—C10'—C11'	70.9 (7)
C3—C4—C5—C6	1.1 (2)	C1—N1—C10'—C11'	−77.9 (7)
C17—O2—C6—C5	−0.3 (2)	C10—N1—C10'—C11'	179.4 (13)
C17—O2—C6—C7	−179.54 (12)	C8—N1—C10'—C15'	−107.9 (5)
C4—C5—C6—O2	−179.17 (13)	C1—N1—C10'—C15'	103.3 (5)
C4—C5—C6—C7	0.0 (2)	C10—N1—C10'—C15'	0.6 (5)
C8—N2—C7—C2	−3.1 (2)	C15'—C10'—C11'—C12'	0.0
C8—N2—C7—C6	177.94 (13)	N1—C10'—C11'—C12'	−178.7 (9)
C3—C2—C7—N2	−176.63 (13)	C10'—C11'—C12'—C13'	0.0
C1—C2—C7—N2	5.9 (2)	C11'—C12'—C13'—C14'	0.0
C3—C2—C7—C6	2.3 (2)	C12'—C13'—C14'—C15'	0.0
C1—C2—C7—C6	−175.14 (12)	C13'—C14'—C15'—C10'	0.0
O2—C6—C7—N2	−3.42 (19)	C13'—C14'—C15'—C16'	−176.7 (13)
C5—C6—C7—N2	177.33 (13)	C11'—C10'—C15'—C14'	0.0
O2—C6—C7—C2	177.57 (12)	N1—C10'—C15'—C14'	178.9 (7)
C5—C6—C7—C2	−1.7 (2)	C11'—C10'—C15'—C16'	176.9 (13)
C7—N2—C8—N1	−1.7 (2)	N1—C10'—C15'—C16'	−4.2 (12)
C7—N2—C8—C9	177.14 (14)	C6—O2—C17—C18	−177.34 (12)
C1—N1—C8—N2	3.5 (2)	O2—C17—C18—C19	−118.44 (14)
C10—N1—C8—N2	−172.17 (13)	O2—C17—C18—C23	63.87 (17)
C10'—N1—C8—N2	−142.4 (4)	C23—C18—C19—C20	0.4 (2)
C1—N1—C8—C9	−175.34 (14)	C17—C18—C19—C20	−177.28 (13)
C10—N1—C8—C9	9.0 (2)	C18—C19—C20—C21	0.2 (2)
C10'—N1—C8—C9	38.8 (4)	C19—C20—C21—C22	−0.4 (2)
C8—N1—C10—C11	−92.72 (14)	C20—C21—C22—C23	0.1 (2)
C1—N1—C10—C11	91.40 (13)	C21—C22—C23—C18	0.5 (2)
C10'—N1—C10—C11	−178.4 (8)	C19—C18—C23—C22	−0.7 (2)
C8—N1—C10—C15	85.66 (14)	C17—C18—C23—C22	176.98 (13)
C1—N1—C10—C15	−90.23 (13)		

### Hydrogen-bond geometry (Å, º)

**Table:**

*D*—H···*A*	*D*—H	H···*A*	*D*···*A*	*D*—H···*A*
C13—H13···O1^i^	0.95	2.54	3.3250 (15)	140
C17—H17*B*···*Cg*1^ii^	0.99	2.62	3.5086 (16)	150
C22—H22···*Cg*1^iii^	0.95	2.77	3.5692 (16)	143

Symmetry codes: (i) −*x*+2, −*y*+2, −*z*+1; (ii) −*x*+1, −*y*, −*z*+1; (iii) −*x*+1, *y*−1/2, −*z*+1/2.
